# Exposure to Silica Nanoparticles Causes Reversible Damage of the Spermatogenic Process in Mice

**DOI:** 10.1371/journal.pone.0101572

**Published:** 2014-07-08

**Authors:** Ying Xu, Na Wang, Yang Yu, Yang Li, Yan-Bo Li, Yong-Bo Yu, Xian-Qing Zhou, Zhi-Wei Sun

**Affiliations:** 1 Department of Health Toxicology and Health Chemistry, School of Public Health, Capital Medical University, Beijing, China; 2 Beijing Key Laboratory of Environmental Toxicology, Capital Medical University, Beijing, China; 3 Department of Laboratory Animal Science, School of Basic Medical Sciences, Capital Medical University, Beijing, China; IIT Research Institute, United States of America

## Abstract

Environmental exposure to nanomaterials is inevitable, as nanomaterials have become part of our daily life now. In this study, we firstly investigated the effects of silica nanoparticles on the spermatogenic process according to their time course in male mice. 48 male mice were randomly divided into control group and silica nanoparticle group with 24 mice per group, with three evaluation time points (15, 35 and 60 days after the first dose) per group. Mice were exposed to the vehicle control and silica nanoparticles at a dosage of 20 mg/kg every 3 days, five times over a 13-day period, and were sacrificed at 15, 35 and 60 days after the first dose. The results showed that **s**ilica nanoparticles caused damage to the mitochondrial cristae and decreased the levels of ATP, resulting in oxidative stress in the testis by days 15 and 35; however, the damage was repaired by day 60. DNA damage and the decreases in the quantity and quality of epididymal sperm were found by days 15 and 35; but these changes were recovered by day 60. In contrast, the acrosome integrity and fertility in epididymal sperm, the numbers of spermatogonia and sperm in the testes, and the levels of three major sex hormones were not significantly affected throughout the 60-day period. The results suggest that nanoparticles can cause reversible damage to the sperms in the epididymis without affecting fertility, they are more sensitive than both spermatogonia and spermatocytes to silica nanoparticle toxicity. Considering the spermatogenesis time course, silica nanoparticles primarily influence the maturation process of sperm in the epididymis by causing oxidative stress and damage to the mitochondrial structure, resulting in energy metabolism dysfunction.

## Introduction

The biological properties of many nanomaterials, coupled with their rapidly expanding productions and usage, have generated concerns that nanoparticles may have unintended impacts when released into natural ecosystems. The applications of silica nanoparticles in industry, biomedicine, food and environmental protection are extremely promising,such as silica nanoparticales are used in drug delivery as carriers because they display good stabilities and excellent biocompatibilities and easy modifications. However, their potential toxic effects remain unclear. Silica nanoparticles have been shown to have more toxicity than silica micron-particles to the male reproductive systems [1], and the smaller the diameter of silica nanoparticles, the greater the toxicity [2]. Silica nanoparticles decreased the number and motility rate of sperm, caused the sperm malformation, and induced the apoptosis of testicle spermatogenic cells in male rats [3], declined male mating rates and live fetus numbers per clutch in rats [4], and decreased the locomotion and reproductive capability of *Caenorhabditis elegans* [5]. The results from histopathological analyses showed that silica nanoparticles led to damage to both Sertoli cells and spermatogenic cells in rats [1]. Li et al. showed that rats exposed to diesel exhaust rich in nanoparticles caused reduction of the weights of both the testis and prostate in male offsprings and disrupted the endocrine activity of the male reproductive system by decreasing the levels of testosterone, progesterone and corticosterone in the serum [6]. However, Ramdhan et al [7] found that the exposure to a low concentration of diesel exhaust rich in nanoparticles increased the levels of plasma testosterone, which contradict to Li et al.'s report [6].

The human male reproductive system is known to be vulnerable to many exogenous materials [8]. Additionally, oxidative stress is known to be one of the main mechanisms of this deterioration [9]. Others and we have attributed silica nanoparticle-induced cytotoxicity to oxidative stress [10]–[12]. However, the effects of silica nanoparticles on the male spermatogenic process remain unclear.

The generation of sperm has a a fixed time period in mice, in which the time course of spermatogenesis (from initial meiosis to sperm release into the seminiferous tubule) is approximately 35 days, and the process of sperm movement from the seminiferous tubule into the epididymis to form mature sperm requires a minimum of approximately 2 weeks [13]. In this study, because of the spermatogenesis time course, we investigated the effects of silica nanoparticles on the spermatogenesis process and its potential mechanism of toxicity in mice, Our study will provide a scientific basis for evaluation the risk of silica particles in the ecosystem to human health.

## Materials and Methods

### Animals and experimental design

Eight-week-old clean grade male ICR mice (Institute of Cancer Research/CD-1) with weights ranging from 20–22 g were obtained from the Beijing Vital River Laboratory Animal Technology Limited Corporation (animal production license number: SCXK2006-0009) (Beijing, China). Groups of 5 mice were raised in a standard plastic cage (26 cm×15 cm×15 cm) with a stainless steel mesh lid in a ventilated room. The mice were maintained at a constant temperature of 20±2°C and a relative humidity of 60±10%, with a 12:12 light/dark cycle. The pad was changed twice per week. All of the mice were provided food and drinking water ad libitum. The animal feed was provided by Beijing Keao Xieli Feedstuff Co., Ltd. The experiments were initiated after a 1-week acclimation period. Before treatment, the mice were fasted overnight.

After 1 week of adaptation to laboratory conditions, 48 healthy adult mice with body weights ranging from 20–23 g were randomly divided into control group and silica nanoparticle group with 24 mice per group, with three evaluation time points (days 15, 35 and 60 after the first dose) per group. Four animals per cage were marked using trinitrophenol. The mice were administered injections of the vehicle control and silica nanoparticles via the tail vein every 3 days, five times over 13 days. Each dose of amorphous silica nanoparticles was 20 mg/kg, and the nanoparticles, which had diameters of 64 nm, were dissolved in physiological saline to obtain a mass concentrations of 12 g/L. Because silica nanoparticales were used as a carrier system in drug delivery as well as getting in blood from environmental exposure, the intravenous administration route of the vehicle injection was selected. The dosage of 20 mg/kg per was based on the previous results of acute toxicity studies [14]. The injection via the tail vein every 3 days, five times over 13 days was according to Bai et al [15], and this schedule was related to human exposures when silica nanoparticales were used as a carrier system in drug delivery. The silica nanoparticles were provided by the School of Chemistry at Jilin University. The mice were sacrificed using 400 mg/kg chloral hydrate at 15, 35 and 60 days after the first dose. Blood, testes and epididymides were collected from each animal for analysis. Plasma samples were obtained from blood by centrifugation at 3500 rpm for 15 min at 4°C. The testicles and epididymides were collected and weighed for the calculation of organ index. The left testes were fixed, and the right testicles and plasma samples were stored at −80°C until analysis. For the fertility assessment, sperm from the epididymides for 6 groups were collected at 15, 35 and 60 days after the first dose.

### Ethics statement

The experiments were strictly conducted in accordance with the institutional guidelines for animal welfare. The protocols were reviewed and approved by the animal experimentation committee of Capital Medical University. All surgery was performed under chloral hydrate, and all efforts were made to minimize suffering.

### Silica nanoparticle preparation and characterization

Silica nanoparticles were prepared using the Stöber method [16]. Briefly, 2.5 mL of tetraethyl orthosilicate (TEOS) (Sigma, USA) was added to a premixed ethanol solution (50 mL) containing ammonia (2 mL) and water (1 mL). The reaction mixture was stored at 40°C for 12 h with continuous stirring (150 r/min). The resulting particles were isolated by centrifugation (12,000 r/min, 15 min), washed three times with physiological saline and then dispersed in 50 mL of physiological saline. The sizes of silica nanoparticles were verified using a transmission electron microscope (TEM) (JEOL JEM2100, Japan), and the size distribution was measured using the Image J software (National Institutes of Health, USA). The hydrodynamic sizes and zeta potential of silica nanoparticles were examined using a Zetasizer (Malvern Nano-ZS90, Britain). Suspensions of silica nanoparticles were dispersed using a sonicator (160 W, 20 kHz, 5 min) (Bioruptor UDC-200, Belgium) before usage to minimize their aggregation.

### Estimation of growth and testis index

The daily weight gain (DWG) was estimated using the following formula: DWG (g/d) = (W2-W1)/T, where *W*1 represent the initial body weight of the mouse, *W*2 represent the weight at 15, 35 or 60 days, and *T* represents the number of days. The testicular index (TI) was estimated using the following formula: TI (%) = testicle weight/body weight ×100%.

### Histological and ultrastructure assessment of testis

The histological structure and ultrastructure of testes were assessed according to description of Wang et al. [10]. Testes were rapidly removed from the mice and fixed in 10% buffered formalin for 24 hours. Testicular tissue was then dehydrated and embedded in paraffin using standard procedures. Sections (4 µm thickness) were deparaffinized and rehydrated. After hematoxylin and eosin (HE) staining, the histopathology of the testis was observed under a fluorescence inverted microscope (Olympus BX53, Japan), and the numbers of spermospores and sperm were counted under the microscope (10×40). Six samples from each group were used for HE staining. Ten fields for every sample were chosen for the counting of the spermospores and sperm. The average numbers of spermospores and sperm from the 10 fields were used as the spermospore and sperm counts for each sample, respectively.

For electron microscopy, the testicles were removed immediately, cut into small pieces, placed in 2.5% glutaraldehyde at 4°C for 2 hours, and washed three times for 10 min each time with phosphate buffer at pH 7.2. The samples were then fixed with 1% osmium tetroxide at 4°C for 2 hours, washed three times for 10 min each time with phosphate buffer at pH 7.2, and dehydrated with ethanol. The dehydrated samples were embedded with EPON 812, cut using an LKB-V microtome, and then stained with 3% uranyl acetate-lead citrate. Testicular ultrastructures were observed using a TEM-2100 transmission electron microscope (JEOL Co., Japan).

### Determination of gonadal hormones in plasma

The free testosterone, luteinizing hormone (LH) and follicle stimulating hormone (FSH) levels in the plasma were determined using ELISA kits provided by Beijing Dongge Biotechnology (Beijing, China) and a Multiskan Ascent Microplate Reader (Thermo Multiskan MK3, USA) at 450 nm.

### Semen evaluation of the epididymis

The sperm concentration, sperm motility rate and the sperm malformation rate were analyzed using the optical microscopy-based hemocytometer method with an appropriate counting chamber as described by Watanabe et al. [17] and using a high-magnification microscope (Leica Microsystems DM1000, USA). The cauda epididymides were quickly placed in a Petri dish with 2 ml of saline and cut into pieces. Then, a small amount of sperm suspension was added to the cell counting plates. The total sperm number and motile sperm number in 4 large cages were counted using a high-magnification microscope (Leica Microsystems DM1000, USA). The sperm concentration = the total sperm number ÷4×10^4^×2; and the sperm motility rate = the motile sperm number ÷ the total sperm number ×100%.

A small amount of sperm suspension was drawn and smeared on a slide, fixed for 10 min with methanol, stained for 1 hour with 1% eosin, and then washed with water. A total of 1000 sperms were counted to determine the proportion of malformed sperm using a high-magnification microscope. The sperm malformation rate = the malformed sperm number ÷1000×100%.

The acrosomal integrity was assessed according to the procedures described by Santos et al [18] and Ozaki et al [19] using a fluorescence inverted microscope (OLYMPUS BX53, Japan). One drop of sperm suspension was placed on a microscopic slide, and through the fluorescence labeling of the sperm nuclei with Hoechst 33258 and acrosomes with FITC–PSA, we examined the integrity of the sperm on days 15, 60 and 90. An overlay of the acrosomes and sperm nuclei showed the integrity of acrosome formation with green acrosomal caps and blue nuclei, whereas only blue nuclei showed incomplete acrosomes. At least 200 sperm were examined for each sample using an fluorescence inverted microscope (OLYMPUS BX53, Japan). Acrosomal integrity (%) = the sperm number with intact acrosomes ÷200×100%.

### Evaluation of sperm fertility in the epididymis

#### Sperm capacitation

The sperm capacitation, which was the process by which a sperm becomes capable of fertilizing an ovum after it reaches the ampullary portion of the uterine tube, was determined according to the method described by Chen et al [20] and Zhang et al [21]. The sperm from the epididymides were collected at 15, 35 and 60 days after the first dose and placed in culture plates with 0.5 ml of M16 culture solution, and paraffin was then added to the mixture. The sperm were transferred to a 5% CO_2_ incubator at 37°C for 15 min,and the sperm concentrations were standardized at concentrations of 5–10×10^5^/ml using M16 culture solution. The sperm were then incubated in a 5% CO_2_ incubator at 37°C for 1 hour to allow sperm capacitation.

#### Collection and cultivation of ova

The collection and cultivation of ovums were performed according to the method described by Nagy et al [22]. On 15, 35 and 60 days after the first dose, 7- to 8-week-old untreated virgin ICR female mice with body weights ranging from 24–26 g (6 mice per group) were given pregnant mare serum gonadotropin (PMSG) at 8 IU per mice by intraperitoneal injection, and after 48 hours, 8 IU human chorionic gonadotropin (HCG) was injected into the peritoneal cavity of each mouse. Fifteen hours after HCG injection, the female mice were killed using the cervical dislocation method, the uterine tube was cut, and the ovarium mound in the ampulla of the fallopian tube was collected using the needlepoint of a 1-ml injector under a stereoscopic microscope. The ovarium mounds were placed in a Petri dish with M16 culture solution, and then cultivated in a 5% CO_2_ incubator at 37°C for 20 min until fertilization.

#### In vitro fertilization

In vitro fertilization was performed according to the method described by Nagy et al [22] and by Chen et al [20]. After sperm capacitation for 1 hour, the capacitated sperm were added to M16 culture solution with ova, and the maximum sperm density was regulated at a concentration of approximately 0.2×10^6^/ml. The mixture was placed in a 5% CO_2_ incubator at 37°C for 6 hours to allow fertilization, the ova were then washed and placed in M2 culture solution, and the bi-pronucleus ova were observed and counted under an inverted microscope (Nikon, PE300). The fertility rate = bi-pronucleus ovum number ÷ the total ovum number ×100%.

### Determination of DNA damage in epididymal sperm

The effect of silica nanoparticles on DNA damage in epididymal sperm was detected by single cell gel electrophoresis (SCGE), which is also known as the comet assay, and the comet experiment kits were provided by Beijing Boletong Biotechnology (Beijing, China). The sperm from the epididymides were collected at 15, 35 and 60 days after the first dose.

Fully frosted microscope slides were prepared with a ‘sandwich’ of normal melting agarose, 10 µl of the sperm suspension (1×10^6^/ml) mixed with 90 µl of low-melting agarose, and a final top layer of low-melting agarose. The slides were immersed in a freshly prepared lysing solution at 4°C for at least 1.5 hours, washed twice with distilled water for 5 min per wash, transferred to a horizontal electrophoresis box containing fresh alkaline electrophoresis buffer, and immersed in buffer for 30 min at 4°C to allow the DNA to unwind. Electrophoresis was performed at 25 V and 300 mA for 30 min at room temperature. The slides were removed, washed three times with neutral buffer for 5 min per wash, and stained with 60 µL of ethidium bromide. The entire procedure was performed in the dark.

Comets representing cellular DNA damage were visualized using a fluorescence inverted microscope (OLYMPUS BX53, Japan) at ×200 magnification. In total, 100 cells were randomly selected for each group, and the degree of DNA damage was quantified. The rate of DNA damage (%) was calculated using the following formula: rate of DNA damage (%) = numbers of comet cells/numbers of all cells counted ×100%.

### Determination of oxidative stress indices in testicular tissue

Testicular homogenates were prepared with saline on ice according to the instructions of a kit provided by the Nanjing Jiancheng Bioengineering Institute (Nanjing, China). The protein concentration of testicle tissue was assayed using a Coomassie Protein Assay Kit. The activities of catalase (CAT), superoxide dismutase (SOD) and glutathione peroxidase (GSH-px), the level of malondialdehyde (MDA) and the ability to inhibit the hydroxyl radical were determined using a Multiskan Ascent microplate reader (Thermo Multiskan MK3, USA) at 405 nm, 550 nm, 412 nm, 532 nm, and 550 nm, respectively.

### Determination of energy metabolism indices in testicular tissue

Testicular homogenates were prepared with saline on ice according to the instructions of the kit provided by the Nanjing Jiancheng Bioengineering Institute (Nanjing, China). The activities of succinate dehydrogenase (SDH) and lactic acid dehydrogenase-C4 (LDH-C4) and the level of adenosine triphosphate (ATP) were determined using a Multiskan Ascent microplate reader (Thermo Multiskan MK3, USA) at 600 nm, 440 nm and 636 nm, respectively.

### Statistical analyses

All of the data were analyzed using the statistical software package SPSS 17.0. The independent sample t-test was used to test differences between the control group and the silica nanoparticles group at the same time point; the chi square test was used to determine both sperm fertility and sperm DNA damage; an analysis of covariance was used to test differences in the daily weight gains and in the testis index throughout the experimental period using the initial body weight as a covariable. All of the values are expressed as the mean values ± standard error (SE). The differences were considered significant at *P*<0.05.

## Results

### The characterization of silica nanoparticles

**Figure 1:**
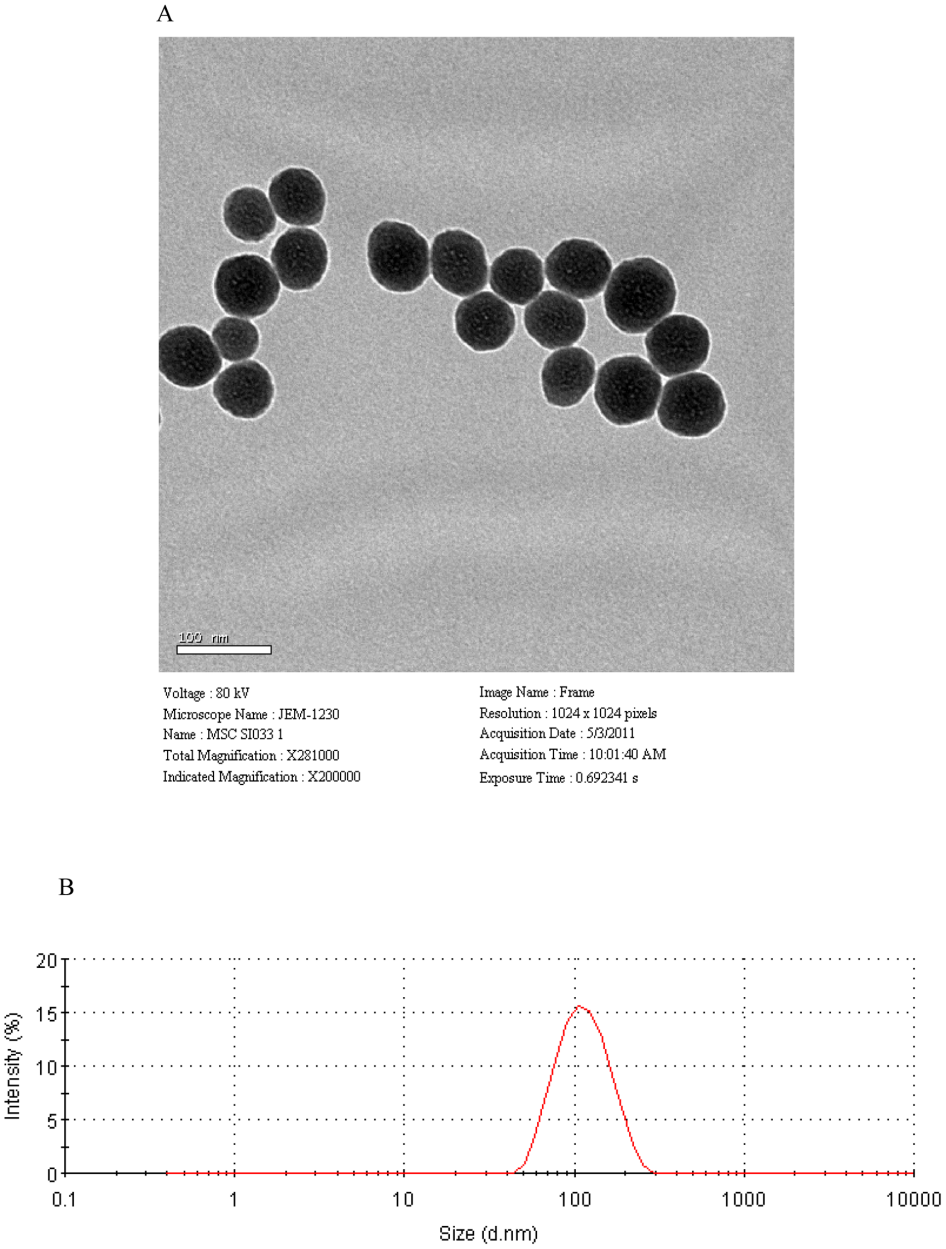
Characterization of silica nanoparticles. (A) Transmission electron microscopy image. (B) Size distribution. Silica nanoparticles exhibited good monodispersity and showed approximately normal distribution.

The TEM images of silica nanoparticles indicated that the particles were spherical in shape with an average diameter of 64.43±10.50 nm (Figure 1A), and their hydrodynamic sizes were measured in physiological saline because the exposure media is 107.5 nm. The nanoparticle distribution was measured using the Image J software, which showed an approximately normal distribution, and the polydispersity index (PDI) was 0.137 (Figure 1B). Our results showed that silica nanoparticles possessed a uniform shape along with a relatively favorable dispersibility in physiological saline. Because of Van der Waals force and hydrophobic interactions with the surrounding media, silica particles generally exhibit ea larger hydrodynamic size in dispersion media than their original size.

### Changes in growth and in the testicular index in mice

On days 15, 35 and 60 in the nanoparticle-treated groups, the average body weights, daily weight gains and the testicular index in silica nanoparticle-treated mice showed no significant differences compared with those values in the control mice (Figures S1A, S1B and S1C) (*P*>0.05); however, the analysis of covariance showed that the daily weight gains were significantly decreased in both the control group and the nanoparticle-treated group over time. On day 15 the daily weight gains were higher than the daily weight gains on day 35, and the daily weight gains on day 35 were higher than the daily weight gains on day 60 (*P*<0.05) (Figure S1B), whereas the testis indices in the nanoparticle-treated and the control groups had no obvious differences (*P*>0.05) between days 15, 35 and 60 (Figure S1C).

### Changes in the testicular tissue structure, the spermatogonium number and the sperm cell number in testicle tissues

The testicular tissue structures in nanoparticle-treated groups exhibited no significant changes compared with control groups on days 15, 35 and 60 after the first dose. The basement membranes of the seminiferous tubules maintained their integrity and were smooth. The layers of the seminiferous epithelium were thick. The regular lumen was filled with mature sperm, and the spermatogenic cells developed normally from spermatogonia to mature sperm cells (Figures S2 A–F).

The spermatogonium number and sperm cell number of the testicle tissues in the nanoparticle-treated mice exhibited no significant changes compared with the control group on days 15, 35 and 60 after the first administered dose (*P*>0.05) (Figures S2G and S2H).

### Changes in the ultrastructure of testicular tissue

**Figure 2:**
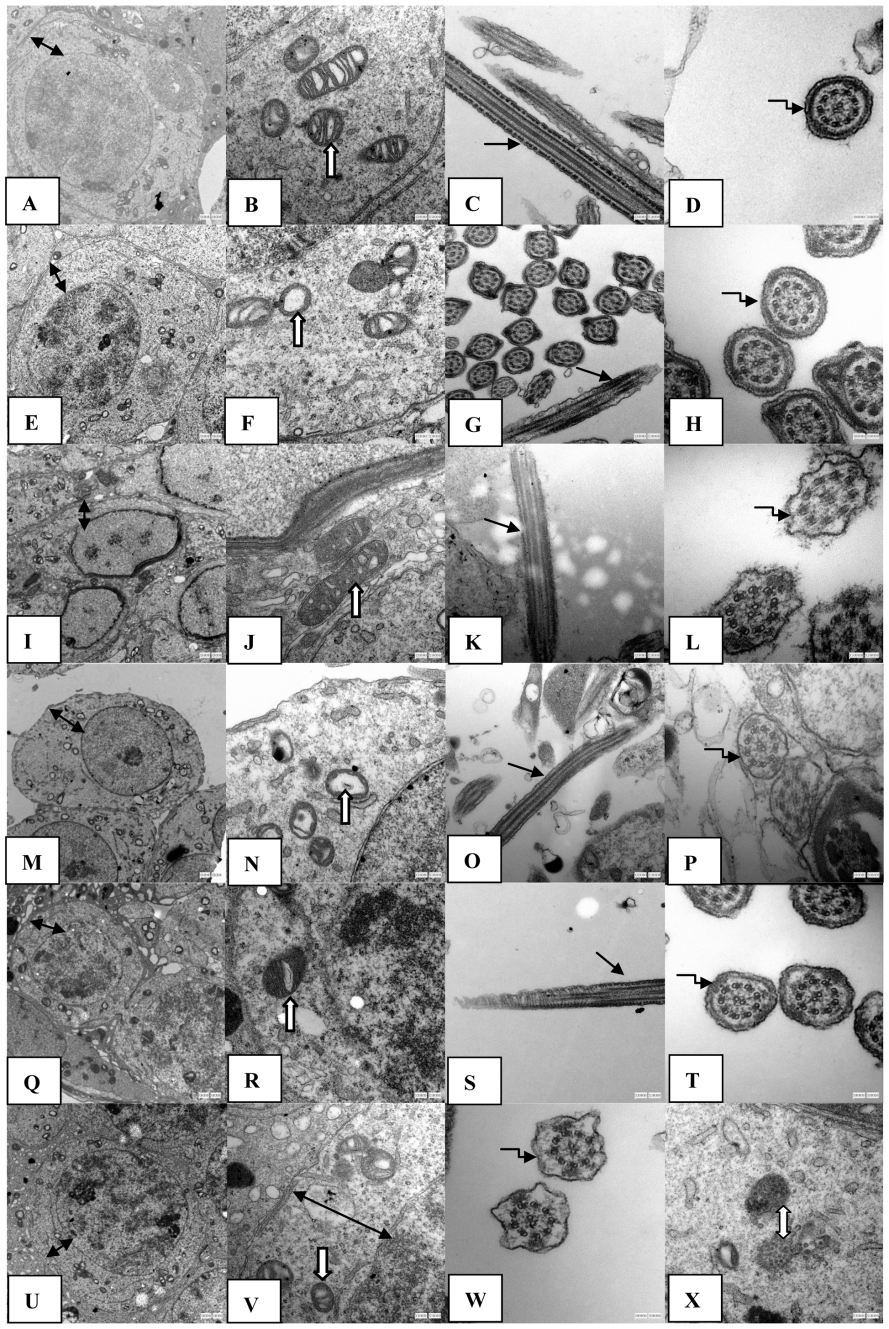
The effects of silica nanoparticles on the ultrastructure of testicular tissue in mice. The silica nanoparticles led to the rupture and even disappearance of the mitochondrial cristae on days 15 and 30 after the first administration of nanoparticles, whereas on day 60 after the first administration of nanoparticles, the mitochondrial structures were better than those on days 15 and 35. The other structures, such as the cell membrane, nuclear membrane and the sperm tail membranes, maintained their integrity on days 15, 35 and 60 after the first administration of nanoparticles. The nanoparticles in spermatogenic cells were encapsulated by organelles in the cytoplasm by day 60 after administering nanoparticles (X). A–X: 15 d control group 6000×(A), 15 d control group30000×(B), 15 d control group 30000×(C), 15 d control group 80000× (D); 15 d Nano-Sio2 group 6000×(E), 15 d Nano-Sio2 group 30000×(F), 15 d Nano-Sio2 group 30000×(G), 15 d Nano-Sio2 group 80000×(H); 35 d control group 6000×(I), 35 d control group 30000×(J), 35 d control group 30000×(K), 35 d control group 80000×(L); 35 d Nano-Sio2 group 6000×(M), 35 d Nano-Sio2 group 30000×(N), 35 d Nano-Sio2 group 30000×(O), 35 d Nano-Sio2 group 80000×(P); 60 d control group 6000×(Q), 60 d control group 30000×(R); 60 d control group 30000×(S), 60 d control group 80000×(T), 60 d Nano-Sio2 group 6000×(U), 60 d Nano-Sio2 group 30000×(V), 60 d Nano-Sio2 80000×(W), 60 d Nano-Sio2 30000×(X). Bidirection black arrow represented cell membrane and nuclear membrane; one-way white arrow represented mitochondrion; one-way black arrow represented sperm tail; elbow shape black arrow represented the cross section of sperm tail; bidirection white arrow represented silica nanoparticle.

On days 15, 35 and 60, the control groups displayed integrity and smoothness of the cell membrane and nuclear membrane (Figures 2A, 2I and 2Q). The outer membranes of the mitochondria were smooth. The inner membranes folded into the mitochondrial cristae were arranged regularly; and the mitochondrial bases were thick (Figures 2B, 2J and 2R). Additionally, the sperm tail membranes were smooth and maintained their integrity, and the cross-sections of the microtubules of the sperm tails showed the “9+2” structure (Figure 2C, 2D, 2K, 2L, 2S and 2T). In contrast, in the nanoparticle-treated group on days 15 and 35, the mitochondrial cristae ruptured and even disappeared, and the mitochondrial base was thin and showed vacuolization. The damage to the mitochondrial cristae on day 35 was larger than that on day 15 (Figures 2F and 2N). However, on day 60, the mitochondrial cristae in the nanoparticle-treated group exhibited mild fractures, and the level of focal vacuolization in the mitochondrial base was less than that on days 15 and 35 (Figure 2V). The other structures, such as the cell membrane, nuclear membrane and the sperm tail membrane, maintained their integrity, and the sperm tail presented clear microtubule structures on days 15, 35 and 60 in the nanoparticle-treated groups(Figure 2E, 2G, 2H, 2M, 2O, 2P, 2U, 2W). In addition, on day 60 in the nanoparticle-treated group, the nanoparticles in spermatogenic cells were encapsulated by organelles in the cytoplasm (Figure 2X), which showed that the silica nanoparticles were mainly accumulated in the perinuclear region, but did not penetrate the nucleus.

### Changes in the levels of gonadal hormones in the plasma and sperm in the epididymis

The levels of testosterone, FSH and LH in the plasma on days 15, 35 and 60 in the nanoparticle-treated groups exhibited no significant differences when compared with their control groups (*P*>0.05) (table 1).

**Table 1:** The effects of silica nanoparticles on the levels of plasma sex hormones in mice (Mean ± S.E.).

Group	Level of testosterone (nmol/L)	Level of follicle stimulating hormone (nmol/L)	Level of luteinizing hormone (nmol/L)
15days control	1.06±0.18	0.41±0.13	137.05±21.84
15days Nano-Sio_2_	0.98±0.05	0.54±0.14	169.05±33.05
35days control	1.06±0.08	0.51±0.20	159.08±8.99
35days Nano-Sio_2_	1.07±0.12	0.59±0.18	153.42±23.92
60days control	0.98±0.08	0.80±0.25	175.59±11.12
60days Nano-Sio_2_	1.07±0.18	0.79±0.30	150.00±22.43

The sperm concentration of the epididymis on day 15 in the nanoparticle-treated group was clearly lower than that of the control group (*P*<0.05), whereas on days 35 and 60 in the nanoparticle-treated groups there were no significant decreases in sperm concentrations when compared with their control groups, although there were decreasing tendencies (*P*>0.05) (table 2).

**Table 2:** The effects of silica nanoparticles on the epididymal sperm in mice (Mean ± S.E.).

Group	Sperm concentration (10^6^/ml)	Sperm abnormity (%)	Sperm motility (%)	Sperm acrosome integrity (%)
15days control	3.04±0.45	13.31±1.99	64.18±7.04	89.83±0.38
15days Nano-Sio_2_	1.20±0.17*	15.69±2.05*	41.61±7.25*	87.21±1.44
35days control	4.35±0.95	13.88±1.85	56.09±3.70	90.25±1.99
35days Nano-Sio_2_	3.03±0.45	15.95±1.40*	26.13±5.76*	86.75±1.09
60days control	3.81±0.55	13.86±2.12	81.56±2.13	89.07±1.90
60days Nano-Sio_2_	3.08±0.50	15.75±0.09	57.71±3.95	91.36±1.71

*Indicates significant difference compared to control group *(P<0.05)*.

The sperm abnormity rates of the epididymis on days 15 and 35 in the nanoparticle-treated groups clearly increased when compared with their control groups (*P*<0.05), whereas on day 60, there was no significant change compared with the control group, although there was an increasing tendency (*P*>0.05) (table 2).

The sperm motility rates of the epididymis on days 15 and 35 in the nanoparticle-treated groups were clearly decreased when compared with the control groups (*P*<0.05), whereas on day 60, there was no significant decrease when compared with the control group (*P*>0.05) (table 2).

The acrosome integrity of sperm in the epididymis had no significant changes on days 15, 35 and 60 days in the nanoparticle-treated groups compared with their control groups (*P*>0.05) (table 2).

The chi square test showed that the epididymal sperm fertility on 15, 35 and 60 days in the nanoparticle-treated groups were not significantly changes when compared with those of the control groups (Figure S3 and Table 3).

**Table 3:** The effects of silica nanoparticles on the sperm fertility of epididymis sperms in male mice.

Group	Number of fertilized ouvm	Number of non-fertilized ouvm	Total number of ouvm	Rate of fertilization (%)	*P* vaule of Chi Square test
15days control	18	21	39	46	0.486
15days Nano-Sio_2_	19	30	49	38	
35days control	16	11	27	59	0.068
35days Nano-Sio_2_	13	23	36	35	
60days control	9	20	29	31	0.931
60days Nano-Sio_2_	9	21	30	31	

### Changes in the DNA damage rate in epididymal sperm

The rate of DNA damage in epididymal sperm on days 15 and 35 in the nanoparticle-treated groups were significantly increased when compared with those of the control groups (*P*<0.05), whereas on day 60, there was no obvious difference between the control group and the nanoparticle-treated group (*P*>0.05) (Table 4 and Figure S4).

**Table 4:** The effects of silica nanoparticles on DNA damage rate of epididymis sperm in male mice.

Group	Number of non-damage sperm	Number of damage sperm	Total number of sperm	Rate of damage sperm (%)	*P* vaule of Chi Square test
15days control	115	17	132	12.88	0.016
15days Nano-Sio_2_	99	32	131	24.43*	
35days control	116	11	127	8.66	0.000
35days Nano-Sio_2_	77	49	126	38.89*	
60days control	129	12	132	9.09	0.424
60days Nano-Sio_2_	116	16	132	12.12	

*Represents significant difference between control group and nanoparticles group at same time *(P<0.05)*.

### Changes in oxidative stress and in energy metabolism in testicular tissue

The MDA level on day 35 in the nanoparticle-treated group was significantly increased when compared with that in the control group (*P*<0.05), whereas on days 15 and 60, there were no obvious changes when compared with those levels in the control groups (*P*>0.05) (Table 5). The ability to inhibit hydroxyl radicals in testicular tissue on days 15 and 35 in the nanoparticle-treated groups were significantly decreased when compared with those of the control groups (*P*<0.05), whereas on day 60, there was no obvious difference between the nanoparticle-treated group and the control group (*P*>0.05) (Table 5). Both the activities of CAT and SOD on days 15, 35 and 60 in the nanoparticle-treated groups exhibited no significant changes when compared with those activities of the control groups (*P*>0.05) (Table 5). The activities of GSH-px on days 15 and 35 also presented no obvious differences when compared with their control groups (*P*>0.05); however, on day 60, the activity of GSH-px was clearly higher than that of the control group (*P*>0.05) (Table 5).

**Table 5:** The effects of silica nanoparticles on the oxidative stress of testicular tissue in mice (Mean ± S.E.).

Group	Malondialdehy (nmol/mg protein)	Ability of inhibiting hydroxylradical (U/mg protein)	Activity ocatalase (U/mg protein)	Activity of supperoxide dismutase (U/mg protein)	Activity of glutathione peroxidase (U/mg protein)
15days control	3.43±0.43	56.74±5.68	14.09±3.82	56.18±4.21	22.45±3.48
15days Nano-Sio_2_	2.54±0.15	44.18±1.91*	10.63±2.29	57.86±7.08	23.17±3.57
35days control	2.48±0.23	48.27±2.13	14.12±2.12	56.38±8.09	23.81±1.91
35days Nano-Sio_2_	4.30±0.54*	33.98±1.74*	11.25±1.41	51.97±6.06	20.08±4.88
60days control	2.31±0.18	39.00±7.05	11.93±3.01	57.92±5.54	23.18±4.49
60days Nano-Sio_2_	1.93±0.08	37.76±3.56	12.61±1.39	61.39±5.93	30.93±5.73*

*Indicates significant difference compared to control group *(P<0.05)*.

The ATP levels in testicular tissue on days 15 and 35 in the nanoparticle-treated groups were obviously decreased when compared with their control groups (*P*<0.05), whereas on day 60, the ATP level exhibited no significant decline (*P*>0.05) (Table 6). The activities of LDH-C4 and SDH in testicular tissue on days 15, 35 and 60 in the nanoparticle-treated groups had no significant differences compared with those activities of the control groups (*P*>0.05) (Table 6).

**Table 6:** The effects of silica nanoparticles on the energy metabolism of testicular tissue in mice (Mean ± S.E.).

Group	ATP level (µmol/g protein)	Activity of lactate dehydrogenase-C4 (U/g protein)	Activity of succinate dehydrogenase (U/mg protein)
15days control	1485.24±140.19	2417.00±291.29	5.37±1.04
15days Nano-Sio_2_	1008.19±147.10*	2610.14±467.46	5.72±1.06
35days control	1507.11±238.82	2581.76±469.67	4.68±0.51
35days Nano-Sio_2_	780.75±193.91*	2604.28±931.15	4.49±0.55
60days control	1053.01±156.13	2184.21±355.43	4.86±1.05
60days Nano-Sio_2_	882.22±124.79	2128.88±399.10	5.56±0.87

*Indicates significant difference compared to control group *(P<0.05)*.

## Discussion

### Effects of silica nanoparticles on the ultrastructure and tissue structure of testicles and on the testicle index

The present results from the testicular ultrastructure showed that the mitochondrial cristae ruptured and even disappeared, and the mitochondrial matrixs were thinner after the first administration of nanoparticles than those of the control groups on days 15 and 35; however, the damage was repaired by day 60. The nanoparticles were encapsulated in vacuoles in the cytoplasm of spermatogenic cells on day 60 in the nanoparticle-treated group. The results indicated that the damages to the testicular ultrastructures that were caused by nanoparticles on days 15 and 35 were repaired and that the nanoparticles were phagocytized by organelles by day 60. Similar to our results, Liu et al. [23] found that approximately 50% of mesoporous hollow silica nanoparticles were removed from the body 4 weeks after their injection in mice. These particles would be excreted from the body, and the entire clearance time of the particles should be greater than 4 weeks. Several reviews suggested that silica nanoparticles were degradable over time in the body [24]–[26].

The present experiment showed that the tissue structures after the first administration of silica nanoparticles exhibited no significant changes compared with the control groups on days 15, 35 and 60, which suggested that silica nanoparticles did not significantly affect the testicle structure. Fan et al [1] found that nano silica could induce histopathological changes in testes. After administering five doses of carbon nanotubes, the thickness of the seminiferous epithelium of the testis decreased at day 15; however, the damage was repaired by days 60 and 90 [15]. Mice that were intratracheally administered carbon-black nanoparticles in 10 doses over 10 weeks also showed partial vacuolation of the seminiferous tubules [15]. The conflicting reason for the above results could be related to the highly diverse structures and properties of nanomaterials and to the multiple exposure methods.

In addition, silica nanoparticles had no significant effects on the growth and on the testis index in mice in the present study. Liu et al found that there were no obvious differences in the body weight at 45 days after the injection of repeated doses of mesoporous hollow silica nanoparticles (MHSNs), whereas the coefficients of the liver and spleen were significantly elevated after injection of 80 mg/kg MHSNs compared with the control group; however, there was no significant difference in the kidneys at this dose in mice [23]. Bai et al [15] found that carbon nanotubes had no significant effects on the average body weights, testis index and epididymis index in nanotube-treated mice, which was similar to our present results. However, Fan et al [1] found that the body weights and testis index in rats clearly decreased when exposed to silica nanoparticles by inhalation. The above results indicated that the growth toxicity of nanoparticles could be related to their dosage used and their properties.

### Effects of silica nanoparticles on the spermatogenic process

In the process of androgone development into mature sperm, the change of any component element could influence development and maturation, leading to morphology abnormalities and hypofunction in sperm. The toxicity of xenobiotics to the male animal reproductive system is primarily associated with obstacles to spermatogenesis and morphological changes of sperm, which are presented through the motility rate, amount and malformation rate of sperm in the epididymis [27]. The present experiment showed that the spermatogonium number and sperm number in testicle tissues had no significant changes compared with the control group on days 15, 35 and 60 after the administration of silica nanoparticles. However, the sperm concentrations in the epididymis on day 15 were clearly decreased when compared with the control groups, whereas on days 35 and 60 there were no significant changes. The sperm abnormity rates in the epididymis significantly increased; however, the motility rates were significantly decreased on days 15 and 35, whereas both rates had no statistical differences on day 60 when compared with their control groups. Our results suggested that silica nanoparticles did not significantly affect the generation abilities of spermatogonia and sperm in the testicles but significantly influenced the quality and quantity in epididymal sperm up to 35 days after exposure, whereas the effects were repaired by day 60.

Sperm are generated in the testes and are transported to the epididymis for concentration and maturation. Spermatogenesis is a prolonged process that spans approximately 35 days, and the sperm maturation in the epididymis is approximately 2 weeks minimum in mice [13]. Considering the spermatogenesis time course, we presume that silica nanoparticles can primarily influence the sperm in the epididymis, whereas there were few effects on androgones and spermatocytes in the testicles, which indicats that the sperm were more sensitive than both spermatogonia and spermatocytes to the toxicity from silica nanoparticles. In addition, the results of the TEM images showed that the nanoparticles were encapsulated by organelles on day 60 in the nanoparticle-treated group, which further supported the above results regarding the recoveries of sperm quality and quantity by day 60.

In addition, the present study showed that the levels of testosterone, FSH and LH in the plasma on days 15, 35 and 60 in the nanoparticle-treated groups had no significant differences when compared with their control groups. The results indicated that the nanoparticles did not influence plasma sex hormone levels under the present exposure conditions. The results from Bai et al. [15] showed that five doses of repeated intravenous injections of carbon nanotubes did not alter the levels of testosterone, LH and FSH in the plasma, which was similar to the present results. However, a more frequent administration of carbon-black nanoparticles (10 doses over 10 weeks) intratracheally elevated the serum testosterone level in mice [28].

### The effects of silica nanoparticles on the fertility and acrosome integrity of sperm in the epididymis

Because What the sperm number in ejaculations per time decreased by 50% could not influence animal fertility, fertility experiments using male animals in vivo are not highly sensitive [29]. The World Health Organization (WHO) Advisory Committees have approved a detection method of human sperm fertilizability in vitro, namely the human sperm zona-free hamster-ovum penetration assay (HOP) [30]. The human sperm is of insemination ability when its penetration rate in HOP is always higher than 10–15%; whereas sperm do not have insemination ability when the penetration rates are always lower than 10% [29]. We collected ova from healthy female mice to be fertilized with the sperm from nanoparticle-treated male mice on days 15, 35 and 60 in vitro. Our results showed that sperm fertility was not affected by the nanoparticles throughout the 60-day period when compared with the control groups. This result is because the acrosome integrity of the sperm was not damaged by silica nanoparticles. We examined the integrity of the sperm on days 15, 35 and 60 after the first administration of silica nanoparticles using fluorescence labeling of sperm nuclei and acrosomes; the results showed that the acrosome integrity of the sperm was not affected by the nanoparticles throughout the 60-day period when compared with their control groups. In mammalian sperm, the acrosome contains digestive enzymes (including hyaluronidase and acrosin) that can break down the outer membrane of the ovum and allow the haploid nuclei in the sperm to join with the haploid nucleus in the ovum [15].

### Damage mechanism of silica nanoparticles on the spermatogenic process

To gain insight into the effects of silica nanoparticles on the mechanisms of the spermatogenic process in mice, we examined the changes in oxidative stress and in energy metabolism. Our results showed that both the activities of CAT and SOD on days 15, 35 and 60 in the nanoparticle-treated groups had no significant changes when compared with the control groups. The activity of GSH-Px on days 15 and 35 had also no obvious differences; however, on day 60, the activity was clearly higher than that of the control group. Although the MDA level on day 35 was significantly increased when compared with the control group, the ability to inhibit hydroxyl radicals was significantly decreased on days 15 and 35 compared with the control group, whereas on day 60 there were no obvious changes. The results suggested that nanoparticles led to oxidative stress in the testes on days 15 and 35. The increase in oxidative stress in the testicles indicated that nanoparticles may harm the male reproductive system but that this system recovers over time (by day 60). The lack of adverse effects on the quality and quantity of the sperm on day 60 in the epididymis further supports this observation. It is likely that the decreases in the ability to inhibit hydroxyl radical and the increase in the MDA level before day 35 of exposure stimulated a compensatory increase in antioxidant enzyme activities, and therefore, the antioxidant enzyme activities had no obvious decline. Similar to our results, carbon nanotubes generated oxidative stress in the testis at day 15; however, the damage was repaired by days 60 and 90 in mice [15]. Fan et al [1] found that nano silica could increase the MDA levels of testes in rats. Our present results showed that nanoparticles induce DNA damage in epididymal sperm on days 15 and 35; however, the damage was repaired by day 60. The results indicated that the oxidative stress induced by silica nanoparticles in testicle tissues could be associated with the damage to the spermatogenic process. Rao et al [31] hypothesized that oxidative stress is one of the mechanisms involved in germ cell apoptosis. Because semen has an extremely weak antioxidant system [32], oxidative stress-mediated DNA fragmentation is common in the spermatozoa of infertile men [33]. Oxidative stress-mediated injury to the male reproductive system is a significant contributing factor in 30–80% of cases of male infertility [9], [34]. Because of some protective mechanisms, such oxidative damage can be kept to a minimum in the liver. However, there is no such protective mechanism in the testes [35]. A disturbed oxidative stress/antioxidant equilibrium caused by toxicants could elevate the oxidative stress level in more vulnerable testes and reduce male fertility [9], [34].

The LDH-C4 activity was closely related to sperm capacitation, movement and sperm-egg binding [36]. SDH is primarily present in the mitochondria of germ cells in the testicles. The present results showed that the activities of LDH-C4 and SDH on days 15, 35 and 60 in the nanoparticle-treated groups had no significant differences compared with control groups. These results support our above results that sperm fertility was not affected by the nanoparticles throughout the 60-day period. The present results also showed that the ATP level in testicular tissue on days 15 and 35 in the nanoparticle-treated groups were clearly decreased, whereas on day 60, there was no significant change when compared with the control group; The present results suggested that nanoparticles can lead to a dysfunction of energy metabolism in the testicles on days 15 and 35; however, this dysfunction recovered over time (by day 60). ATP level directly reflects the mitochondrial functional state [37]. The testicular ultrastructure results from our experiment showed that the mitochondrial cristae ruptured and even disappeared on days 15 and 35 in the nanoparticle-treated groups; however, the damage was repaired by day 60, which supports our above results of ATP level changes. Our results indicated that the energy metabolism dysfunction caused by silica nanoparticles in testicle tissues is involved in the damage to the spermatogenic process. Nanoparticles cand influence the energy metabolism by damaging the mitochondria structure and, therefore, decreased the ATP level. The damaged mitochondrial structure could affect cellular energy production, leading to an increase in the malformation of sperm due to energy deficiency, which results in a decrease in sperm quantity and quality on days 15 and 35. Sperm tails are the movement organs of sperm, and sperm movement requires not only structural integrity but also a normal energy supply. Mitochondrial energy metabolism dysfunction may be the main reason for the observed decrease in the sperm motility rate. With nanoparticles excreted from the body or encapsulated by organelles, oxidative stress vanishes, and the above damages were all repaired/recovered by day 60.

## Conclusions

In conclusion, because of the spermatogenesis time course, the study suggests that: (1) silica nanoparticles primarily affect the maturation process of sperm in the epididymis; the sperm in the epididymis are more sensitive to the toxicity of silica nanoparticles than both spermatogonia and spermatocytes. (2) Nanoparticles lead to the reversible damage of epididymal sperm without affecting fertility. (3) The nanoparticles can damage the quantity and quality of sperm in the epididymis by causing oxidative stress and by damaging the structure of mitochondria, resulting in energy metabolism dysfunction.

## Supporting Information

Figure S1 **The effects of silica nanoparticles on the body weight (A), daily increase (B) and testicular index in mice (Mean ± S.E.).** Silica nanoparticles had no significant effects on the average body weights, daily weight gains and the testicular index in silica nanoparticle-treated mice (Figure **S1**A, S1B and S1C) on days 15, 35 and 60 after the first administration of nanoparticles; however, significantly decreased the daily weight gains (Figure S1B). The values with different capital superscripts and lowercase letters are significantly different among different time control groups and nano-silica groups (p<0.05).
(TIF)

Figure S2 **Pathologic and morphometric analyses of testes in mice treated with silica nanoparticles.** The testicular tissue structures including the basement membranes of seminiferous tubules and the layers of the seminiferous epitheliums in nanoparticle groups had no significant changes when compared with control groups on days 15, 35 and 60 after the first dose (Figure S2 A–F). A–H: 15 d control group 400×(A), 15 d silica group 400×(B), 35 d control group 400×(C), 35 d silica group 400×(D), 60 d control group 400×(E), 60 d silica group group 400×(F), spermatogonium number (G) and sperm cell number (H). The values with completely different superscript letters are significant different among nano-silica groups (p<0.05).
(TIF)

Figure S3 **The images of fertilized ovum and non-fertilized ovum** ***in vitro*** **.** A–B: Fertilized ovum(A), Non-fertilized ovum (B).
(TIF)

Figure S4 **Single cell gel electrophoresis (SCGE) images of epididymal sperm in male mice on 15, 35 and 60 days after given silica nanoparticles.** Slica nanoparticles significantly increased the rate of DNA damage in epididymal sperm on days 15 and 35 after administration of nanoparticles, whereas on day 60, there was no obvious difference between the control group and the nanoparticle-treated group. The green arrow points to DNA damage.
(TIF)
