# Supplementary figures and images for: Exposure to Silica Nanoparticles Causes Reversible Damage of the Spermatogenic Process in Mice
Source: PLoS One. 2014 Jul 8;9(7):e101572. doi: 10.1371/journal.pone.0101572 (PMC4086902; doi:10.1371/journal.pone.0101572)

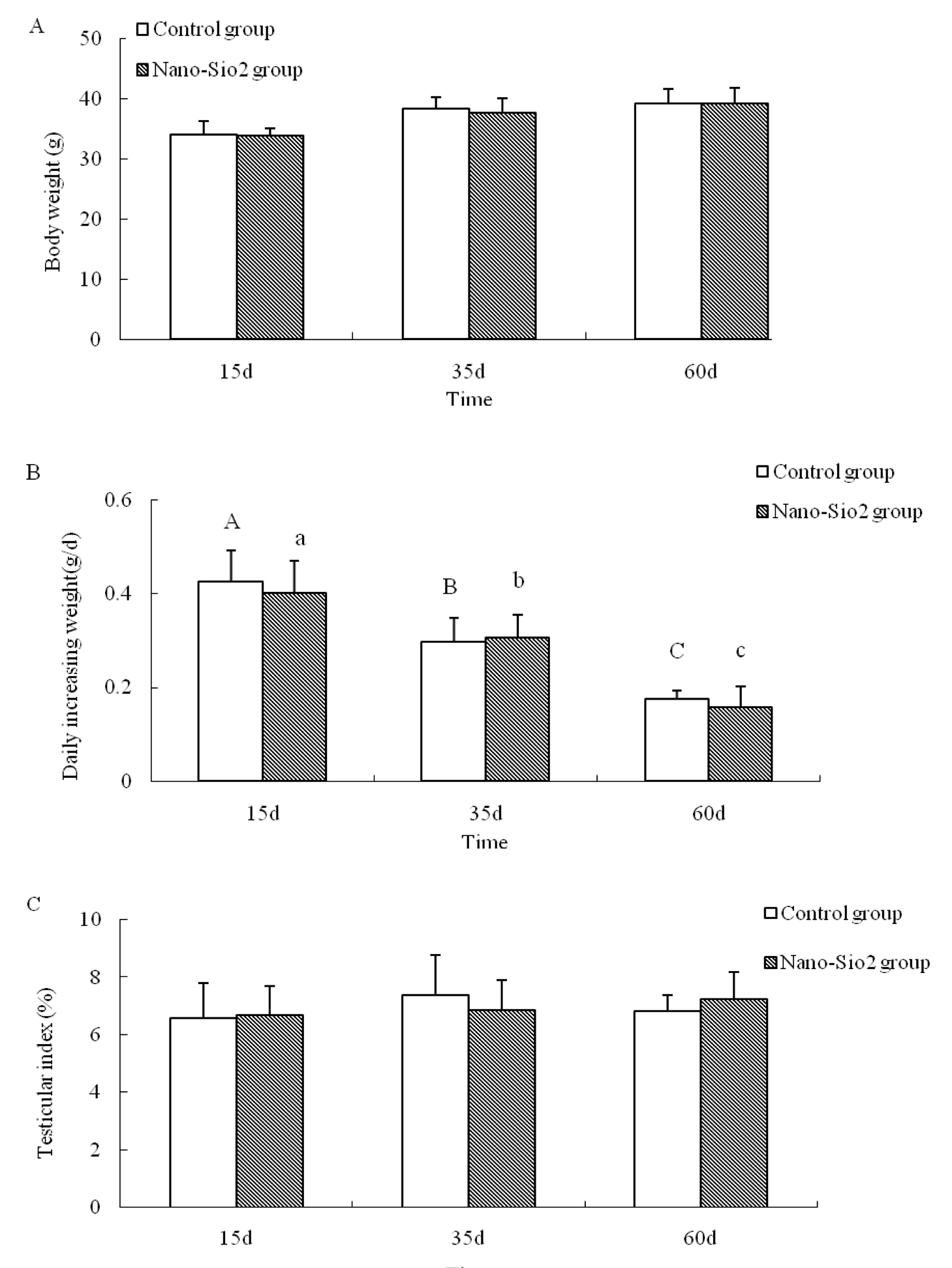

Supplement: Figure S1 — The effects of silica nanoparticles on the body weight (A), daily increase (B) and testicular index in mice (Mean ± S.E.). Silica nanoparticles had no significant effects on the average body weights, daily weight gains and the testicular index in silica nanoparticle-treated mice (Figure S1A, S1B and S1C) on days 15, 35 and 60 after the first administration of nanoparticles; however, significantly decreased the daily weight gains (Figure S1B). The values with different capital superscripts and lowercase letters are significantly different among different time control groups and nano-silica groups (p<0.05). (TIF) [file pone.0101572.s001.tif]

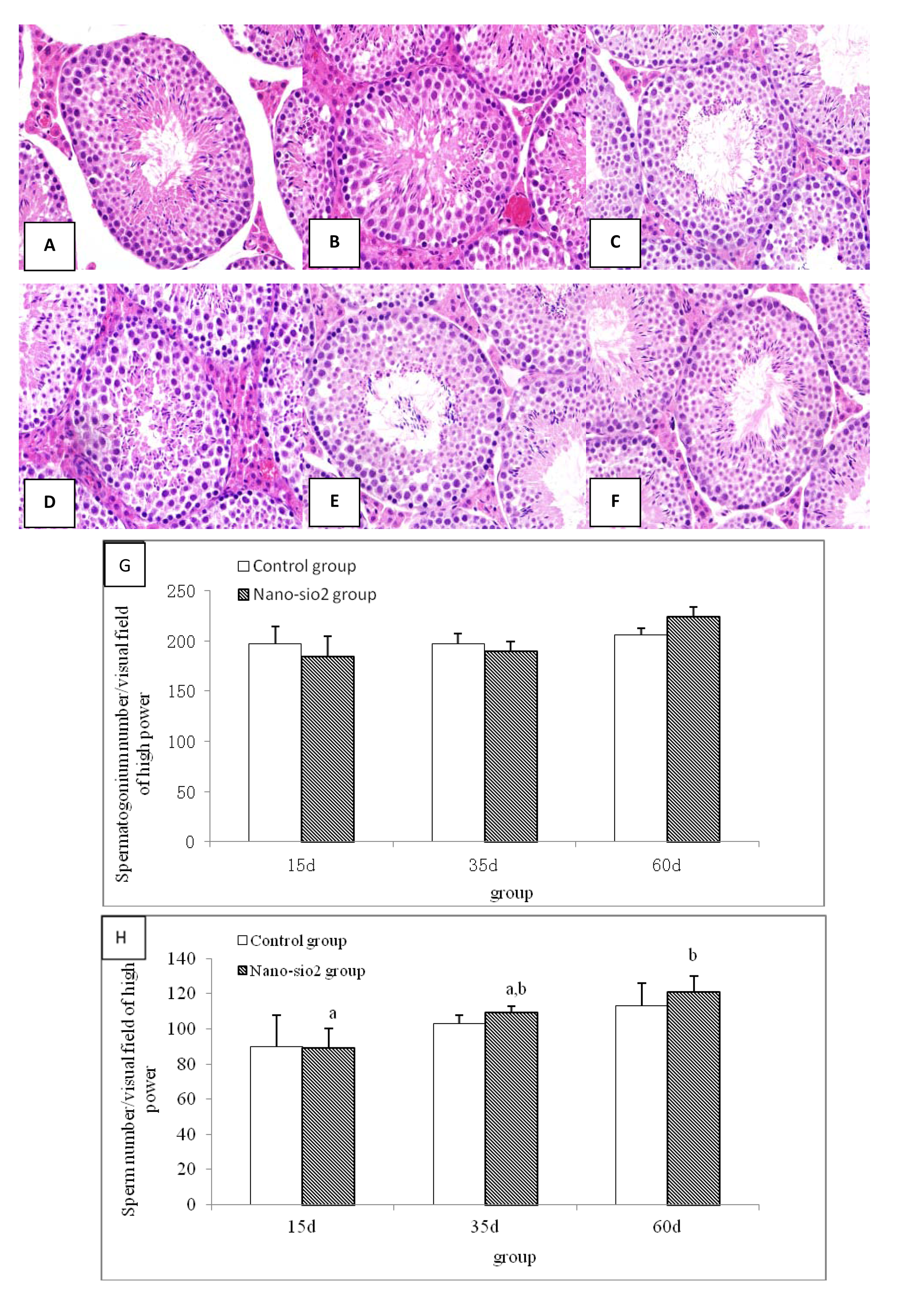

Supplement: Figure S2 — Pathologic and morphometric analyses of testes in mice treated with silica nanoparticles. The testicular tissue structures including the basement membranes of seminiferous tubules and the layers of the seminiferous epitheliums in nanoparticle groups had no significant changes when compared with control groups on days 15, 35 and 60 after the first dose (Figure S2 A–F). A–H: 15 d control group 400×(A), 15 d silica group 400×(B), 35 d control group 400×(C), 35 d silica group 400×(D), 60 d control group 400×(E), 60 d silica group group 400×(F), spermatogonium number (G) and sperm cell number (H). The values with completely different superscript letters are significant different among nano-silica groups (p<0.05). (TIF) [file pone.0101572.s002.tif]

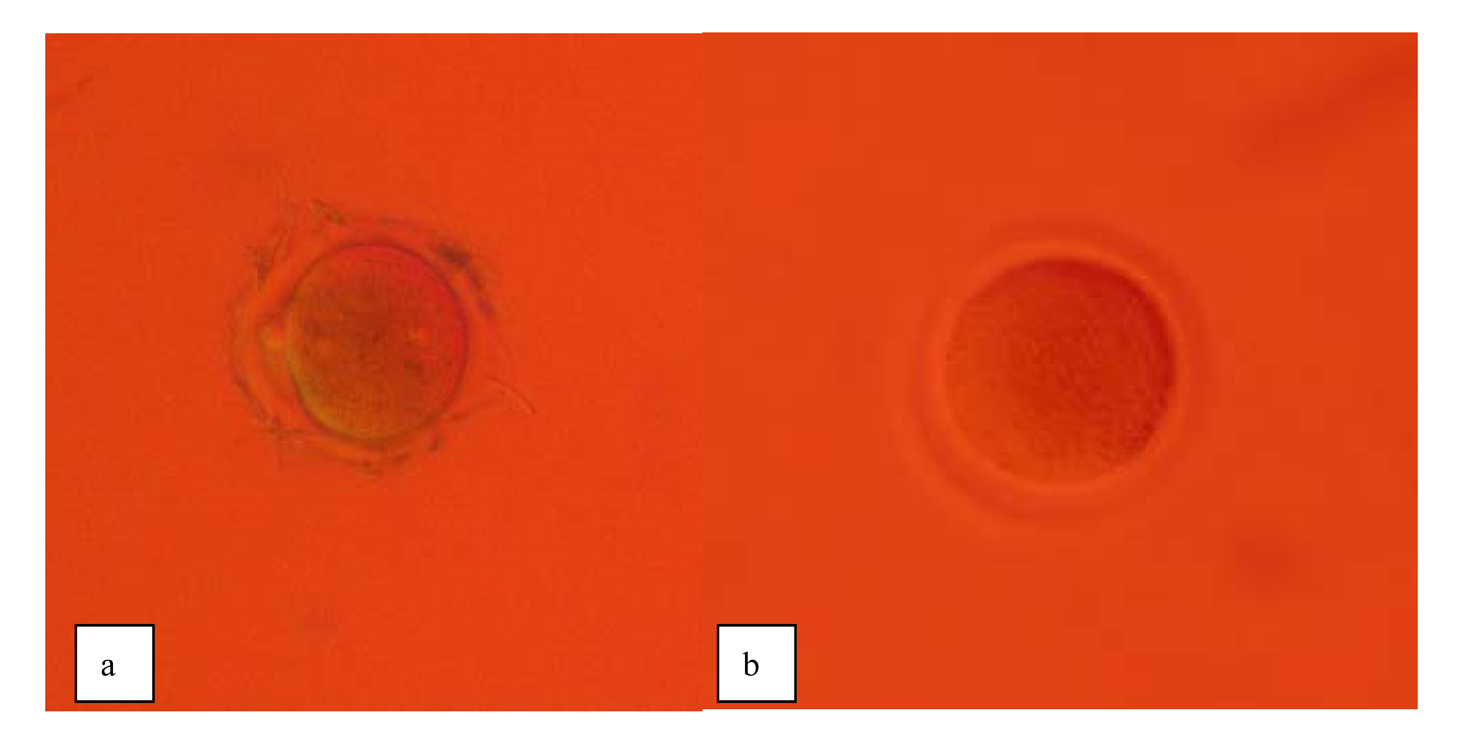

Supplement: Figure S3 — The images of fertilized ovum and non-fertilized ovum in vitro . A–B: Fertilized ovum(A), Non-fertilized ovum (B). (TIF) [file pone.0101572.s003.tif]

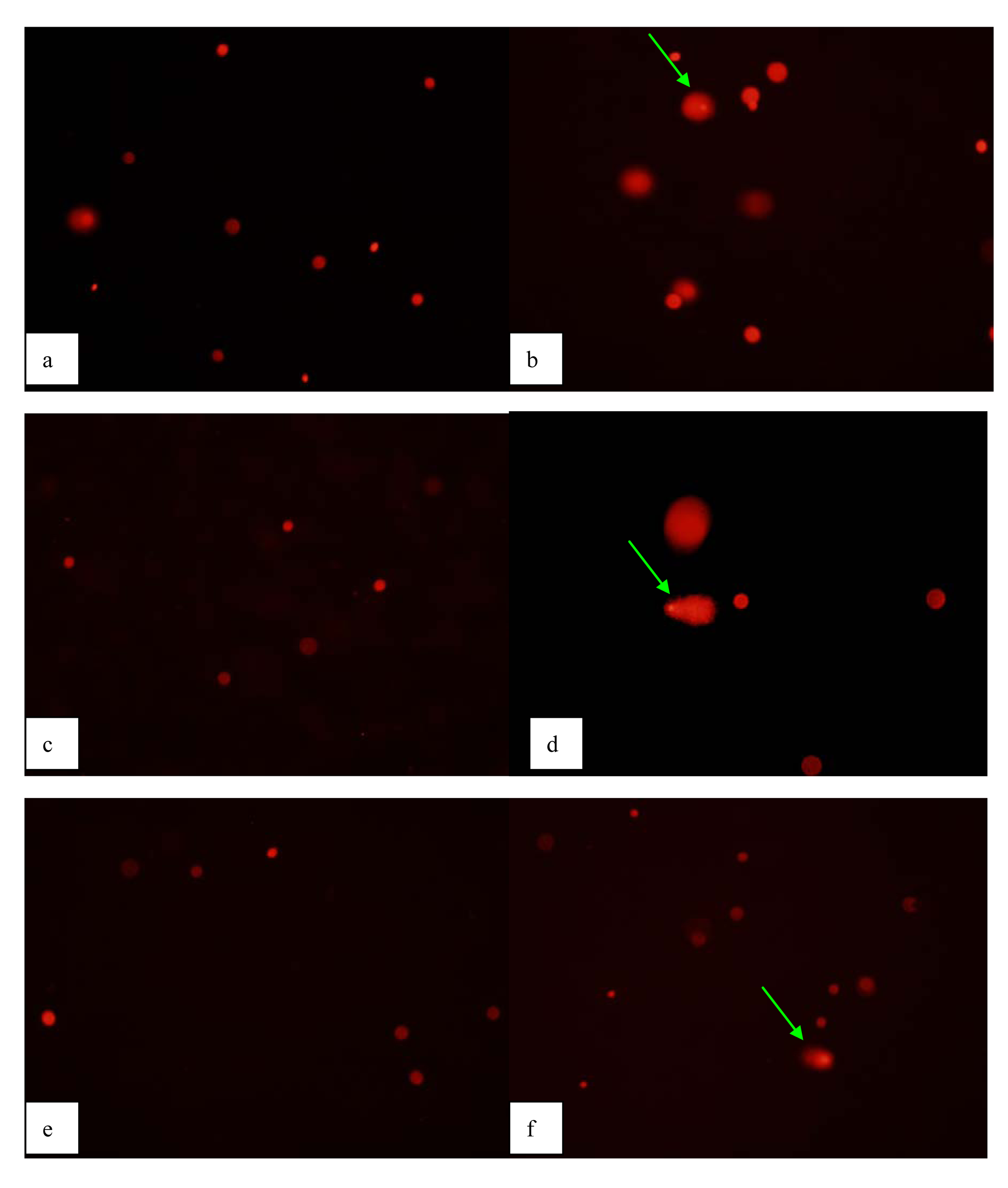

Supplement: Figure S4 — Single cell gel electrophoresis (SCGE) images of epididymal sperm in male mice on 15, 35 and 60 days after given silica nanoparticles. Slica nanoparticles significantly increased the rate of DNA damage in epididymal sperm on days 15 and 35 after administration of nanoparticles, whereas on day 60, there was no obvious difference between the control group and the nanoparticle-treated group. The green arrow points to DNA damage. (TIF) [file pone.0101572.s004.tif]
